# Large-scale polymorphism discovery in macaque G-protein coupled receptors

**DOI:** 10.1186/1471-2164-14-703

**Published:** 2013-10-11

**Authors:** Dharmendra B Goswami, Lisa M Ogawa, Joshua M Ward, Gregory M Miller, Eric J Vallender

**Affiliations:** 1 New England Primate Research Center, Harvard Medical School, One Pine Hill Drive, Southborough, MA 01772, USA

**Keywords:** Resequencing, Single-nucleotide polymorphism, Indian-origin rhesus macaques, Chinese-origin rhesus macaques, Cynomolgus macaques

## Abstract

**Background:**

G-protein coupled receptors (GPCRs) play an inordinately large role in human health. Variation in the genes that encode these receptors is associated with numerous disorders across the entire spectrum of disease. GPCRs also represent the single largest class of drug targets and associated pharmacogenetic effects are modulated, in part, by polymorphisms. Recently, non-human primate models have been developed focusing on naturally-occurring, functionally-parallel polymorphisms in candidate genes. This work aims to extend those studies broadly across the roughly 377 non-olfactory GPCRs. Initial efforts include resequencing 44 Indian-origin rhesus macaques (*Macaca mulatta*), 20 Chinese-origin rhesus macaques, and 32 cynomolgus macaques (*M. fascicularis*).

**Results:**

Using the Agilent target enrichment system, capture baits were designed for GPCRs off the human and rhesus exonic sequence. Using next generation sequencing technologies, nearly 25,000 SNPs were identified in coding sequences including over 14,000 non-synonymous and more than 9,500 synonymous protein-coding SNPs. As expected, regions showing the least evolutionary constraint show greater rates of polymorphism and greater numbers of higher frequency polymorphisms. While the vast majority of these SNPs are singletons, roughly 1,750 non-synonymous and 2,900 synonymous SNPs were found in multiple individuals.

**Conclusions:**

In all three populations, polymorphism and divergence is highly concentrated in N-terminal and C-terminal domains and the third intracellular loop region of GPCRs, regions critical to ligand-binding and signaling. SNP frequencies in macaques follow a similar pattern of divergence from humans and new polymorphisms in primates have been identified that may parallel those seen in humans, helping to establish better non-human primate models of disease.

## Background

Animal research has provided the scientific community with extraordinary advances in medicine from the development of vaccines to the prevention and treatment of diseases. Unfortunately at present 85% of novel therapeutics fail in preclinical and early phase clinical trials and of the therapies that reach late phase trials an additional 50% fall short due to an inability to demonstrate efficacy and safety [1]. Reasons for these shortcomings include low patient recruitment, poor study design, and ineffective use of animal models [1,2]. Coupled with soaring drug development costs including both financial commitments and in years of labor, these shortfalls necessitate a biological and economic need for fundamental changes in the bench to bedside process. Furthermore, with advances in genome sequencing technologies there is a growing awareness that animal models fall short in terms of predictive power. A recent study comparing the genomic responses of human inflammatory diseases to mouse models, for example, suggested that mice poorly mimic the human genetic response [3]. Continued progress in the understanding of human disease pathologies and the development of safe and effective therapies demands a more comprehensive understanding of animals in preclinical research.

Although greater numbers of rodents are used in biomedical research, non-human primates are the gold standard of animal models in preclinical research offering advantages which include greater similarities in genome organization and sequence, behavior, and physiology [4]. The rhesus (*Macaca mulatta*) and cynomolgus (*M. fascicularis*) macaque are two of the most commonly used non-human primate species in research laboratories, sharing ~93.5% of their genome with humans [5]. In academic research non-human primate use is most common in the fields of microbiology (HIV/AIDS), biochemistry/pharmacology, and neuroscience [6]. Because of similarities in physiology and the central nervous system, non-human primates, for example, are crucial in stem cell-based regenerative medicine to ensure the efficacy and long-term safety of autologous cell therapies, which is not possible in rodents [7]. In industry settings, non-human primates are important to drug development and are commonly found in drug metabolism and toxicology studies [8,9]. Despite these distinct advantages, drawbacks to non-human primates include greater genetic heterogeneity and higher costs which tend to lead, in turn, to small samples sizes [4]. Ultimately these disadvantages contribute to the limited use of non-human primates in biomedical research, particularly in academic settings. This necessitates the need to optimize study design through careful animal selection, which can only be accomplished by gaining a more thorough understanding of the genetic variation inherent in non-human primates and more specifically the functional effects relative to similar variation in humans.

Comparative genetic studies between non-human primates and humans have increased from early candidate gene studies through whole genomes, with limited but significant research now focusing on variation within species. Candidate polymorphism studies in non-human primates, for example, have revealed variation in the dopamine transporter (*DAT*) [10,11], tryptophan hydroxylase 2 (*TPH2*) [12,13], the serotonin transporter (*SLC6A4*) [14-18], monoamine oxidase A (*MAOA*) [17,19], brain-derived neurotrophic factor (*BDNF*) [20], neuropeptide Y (*NPY*) [21], and corticotropin-releasing factor (*CRH*) [22] that parallel and functionally mimic variation found in humans. In addition, not only are similar effects seen when these polymorphisms are compared *in vitro* but similar associations to organismal phenotypes also persist across human and non-human primate species.

G-protein coupled receptors (GPCRs) comprise the largest family of cell surface receptors. Though they share a similar seven transmembrane domain structural homology, they are extraordinarily diverse with the capacity to transduce messages triggered by ligands as varied as photons, organic odorants, nucleotides, nucleosides, peptides, lipids and proteins [23]. Consequently, excluding the olfactory subgenome, which represents a distinct class of GPCRs with targeted function [24,25], this receptor superfamily represents the largest group of druggable targets [26] comprising >50% of pharmacotherapies on the market today. Interestingly, only a third of these GPCRs have been explored for drug development portending a future active area of research for the discovery of novel therapeutics [26,27]. Polymorphisms in GPCRs however can affect drug efficacy through altered ligand binding, receptor activation/inactivation, and/or varied signaling cascades. Characterizing non-human primate variation in GPCRs can therefore complement the study of disease and pharmacotherapies whilst refining the translational capacity of non-human primates in preclinical research.

Here the exonic sequence of non-olfactory GPCRs in 44 Indian-origin rhesus, 20 Chinese-origin rhesus, and 32 cynomolgus macaques was resequenced to gain a better understanding of the natural variation in GPCRs of common non-human primate models. Polymorphisms were then compared to fixed species differences and similar variation in humans. Predicted and known protein structural features were also used to better contextualize the changes and their likely functional effects. Comprehensive polymorphism data in non-human primates not only will facilitate characterization of functional variation at important drug targets and support a better understanding of disease but will also aid in informed *a priori* selection of animals in preclinical studies and increased translational validity of the non-human primate models ultimately leading to more safe and effective pharmacotherapies and treatments.

## Results and discussion

Over 700 million reads were generated representing over 35 billion base pairs of sequence from 96 animals. The number of reads per animal ranged from approximately 1 million to 10 million with a median of just over 6.5 million. These reads were aligned to the rhesus genome with the percentage of reads mapped confidently ranging from a minimum of 91.8% to a maximum 95.6%, with a median of 94.3%. Of the 377 GPCRs targeted, 354 had complete coverage across the gene. For the remainder, most had localized failures, often a single missing exon or portion of an exon, due to poor or inadequate annotation in the rhesus genome. It is probable that RNA-based approaches or improved annotation would ameliorate many of the failures. While there were 8 animals for which more than 20% of regions were not called, presumably due to suboptimal DNA quality or some other manual error in the processing stages, the median coverage for individual animals was 99.75%.

**Figure 1:**
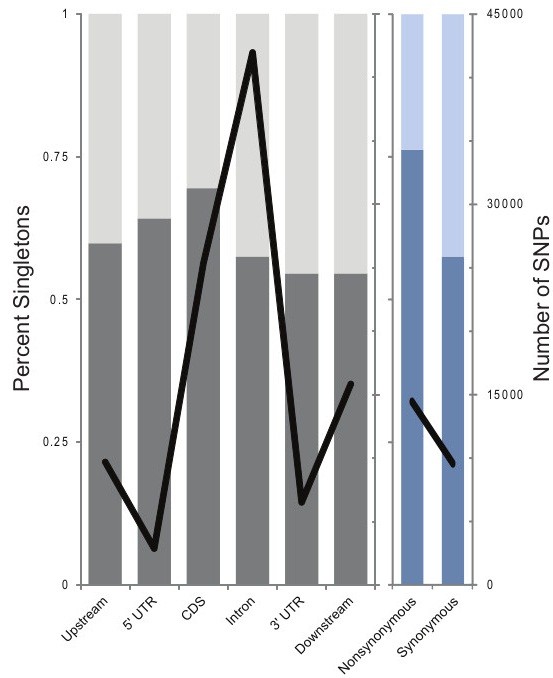
**SNP annotation by category.** Singletons are light and SNPs found in multiple individuals are dark. Line graph shows number of SNPs identified.

Over 100,000 SNPs were identified across all regions and populations (Figure 1, Additional file 1: Table S1). Although the DNA capture targeted exons, a large proportion of adjacent introns, upstream, and downstream flanking regions were also resequenced. Within exons, coding regions were the primary focus, though polymorphisms were also found in the 5′ and 3′ untranslated regions (UTRs) in large numbers. It is worth noting, however, that 3′ UTRs, in particular, may be poorly annotated in the rhesus genome and difficult to comprehensively interrogate. In coding sequence, nearly 25,000 coding SNPs were identified including over 14,000 non-synonymous and over 9,500 synonymous SNPs. As expected, regions showing the least evolutionary constraint show greater rates of polymorphism and greater numbers of higher frequency polymorphisms. Across non-coding regions, with the notable exception of the 5′ UTR, singletons represent roughly 60% of all polymorphisms. Synonymous polymorphisms within coding regions are also at 61.2%. In comparison, non-synonymous polymorphisms show a much greater proportion of singletons, 81.6%, consistent with a slightly deleterious genetic load. The 5′ UTR shows an intermediate proportion of singletons, 67.8%, perhaps reflective of greater constraint due to a higher density of regulatory elements.

While much fewer, frameshift and nonsense (stop gain) mutations in coding sequence were also observed. For the most part these were rare events (Table 1). 83% (38/47) of frameshift mutations were observed in a single individual and nearly 96% (1,049/1,098) of nonsense mutation were singletons. Among common mutations (defined herein as mutations observed in multiple individuals) private alleles predominated. One note of caution, however, in that annotation difficulties within the rhesus genome may have overinflated these numbers. Because of the relative likelihood that these mutation will result in functional effects, often creating natural knockouts, particularly common mutations were further examined (Table 2). Of note, is that five of the thirteen most common of these variants all occur in the *CELSR1* gene, notable for its extensive N-terminal domain. This and other variation offers fertile ground for potential animal model development going forward.

**Table 1:** Frameshift and stop gain mutations

	**Frameshift**	**Stop gain**
**Singleton**	39	1049
**Common**	8	49
*Chinese Rhesus*	1	6
*Indian Rhesus*	1	16
*Cynomolgus*	0	11
*Chinese-Indian*	2	4
*Chinese-Cynomolgus*	1	0
*Indian-Cynomolgus*	0	12
*All*	3	0

Common alleles defined as those observed in multiple individuals.

**Table 2:** High frequency frameshift and stop gain mutations

						**Chinese rhesus**			**Indian rhesus**			**Cynomolgus**		
**Position**	**Reference allele**	**Allele X**	**Allele Y**	**Gene**	**CDS consequence**	**n**	**X**	**Y**	**n**	**X**	**Y**	**n**	**X**	**Y**
chr1:112314147	-	-	g	CELSR2	Frameshift	21	76.2%	23.8%	35	80.0%	20.0%	30	65.0%	35.0%
chr10:90409612	g	g	a	CELSR1	Stop gain	20	70.0%	30.0%	30	76.7%	23.3%	29	100.0%	0.0%
chr1:84773138	a	a	-	LPHN2	Frameshift	20	77.5%	22.5%	16	84.4%	15.6%	29	84.5%	15.5%
chr3:196105681	c	c	t	VIPR2	Stop gain	21	100.0%	0.0%	33	100.0%	0.0%	30	76.7%	23.3%
chr14:53770846	c	c	t	MRGPRX3	Stop gain	21	95.2%	4.8%	31	82.3%	17.7%	30	100.0%	0.0%
chr10:90386626	g	g	a	CELSR1	Stop gain	20	100.0%	0.0%	26	100.0%	0.0%	29	77.6%	22.4%
chr13:124076459	c	c	t	HTR5A	Stop gain	14	100.0%	0.0%	13	100.0%	0.0%	20	70.0%	30.0%
chr1:9594364	c	c	t	TAS1R1	Stop gain	21	100.0%	0.0%	33	100.0%	0.0%	29	84.5%	15.5%
chr10:90412270	g	g	a	CELSR1	Stop gain	21	100.0%	0.0%	30	100.0%	0.0%	29	84.5%	15.5%
chr3:95248643	c	c	t	GHRHR	Stop gain	17	79.4%	20.6%	21	100.0%	0.0%	28	100.0%	0.0%
chr10:90411842	g	g	a	CELSR1	Stop gain	21	85.7%	14.3%	28	98.2%	1.8%	29	100.0%	0.0%
chr10:90385309	g	g	a	CELSR1	Stop gain	20	85.0%	15.0%	24	100.0%	0.0%	29	100.0%	0.0%
chr14:86696825	t	t	-	GRM5	Frameshift	19	97.4%	2.6%	27	98.1%	1.9%	28	92.9%	7.1%

### Population demography

**Figure 2:**
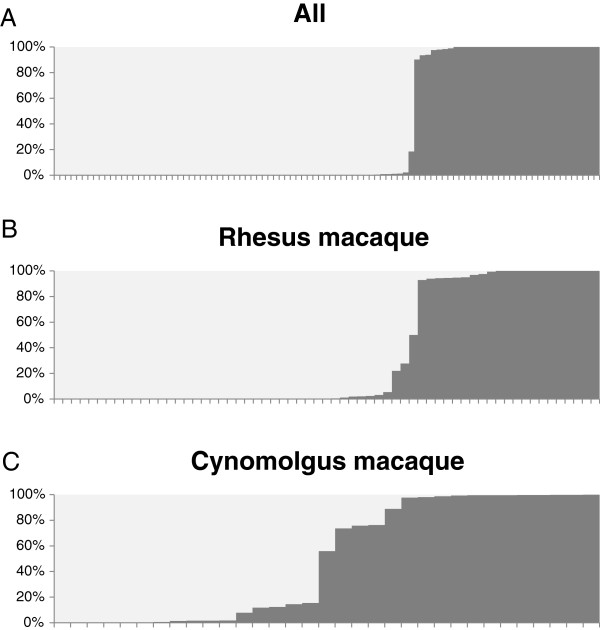
**STRUCTURE analyses of populations using GPCR polymorphisms.** Each individual is represented by a column and inferred ancestry is shown by color. **A.**, k = 2 analysis separating rhesus from cynomolgus macaques. **B.**, k = 2 analysis separating Chinese- and Indian-origin rhesus macaques, note the hybrids. **C.**, k = 2 analysis on cynomolgus macaques identifies what appears to be cryptic substructure.

Cynomolgus and rhesus macaques, despite being separate species, share polymorphisms [28] and may show some evidence of natural admixture [29]. Both cynomolgus macaques and rhesus macaques are widely distributed across southeast Asia and cryptic population substructure has been a pervasive problem in biomedical research. In Indian- and Chinese-origin rhesus differences in susceptibility and progression of simian immunodeficiency virus (SIV) as a model of HIV/AIDS are the most recognized confounds in research laboratories [30,31] though other behavioral and physiological differences also certainly exist [32-37]. Using STRUCTURE [38], rhesus and cynomolgus macaques were readily separated (Figure 2A). It is perhaps noteworthy that those animals that are less unambiguous are those for which fewer reads were generated and had lower levels of coverage across genes. When only rhesus macaques were considered (Figure 2B) the Indian and Chinese subpopulations readily separated, though three putative Indian-origin animals showed significant proportions of Chinese admixture, one a 50/50 hybrid and two 75/25 hybrids. During retrospective investigation these animals were confirmed as known hybrids of the inferred proportions. Indian-origin rhesus macaques were sourced from three locations (New England Primate Research Center, Oregon National Primate Research Center, Caribbean Primate Research Center) but no genetic subdivision was observed. With regards to the cynomolgus macaques, although all of the individuals used in this study were derived from Mauritius stock, unexpected cryptic substructure was observed (Figure 2C). This substructure remains unexplained though recent published studies have indicated similar uncertainty as to the genetic homogeneity of the population [39]. In any case, further study and consideration is warranted.

**Figure 3:**
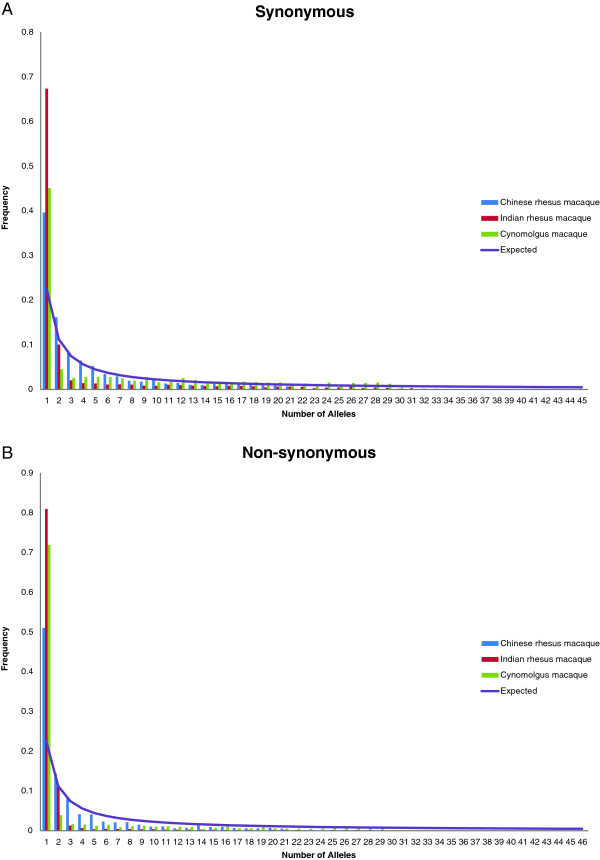
**Frequency distribution of polymorphisms in the three populations. A.** Synonymous changes. **B.** Non-synonymous.

The demographic history of the subgroups can be confirmed by comparing the allele frequency spectra. As predicted by population genetics theory, the vast majority of these SNPs [8] are singletons. In fact, singletons are overrepresented in all three populations (counting the cynomolgus macaques as a single panmictic population) suggestive of recent population expansion (Figure 3A-B). Again, however, cryptic population substructure in Mauritian cynomolgus macaques is supported by an excess of high frequency alleles with a corresponding decline in mid-frequency alleles. While the two populations of rhesus macaques behave similarly, the allele frequency spectrum of the Chinese population appears more similar to that expected under neutrality while the Indian population appears to have undergone a more recent population expansion. These findings are contrary to conventional understandings of the population history of rhesus macaques and to previous genetic studies [40]. It is possible that this discrepancy can be explained through greater artificial selection by humans as the Indian rhesus macaques have been bred in biomedical research facilities under strong pressures to avoid inbreeding and to maximize genetic diversity, while Chinese populations are more recently derived from wild caught animals. It is also possible that cryptic differential natural selective regimes otherwise exist between the populations. As expected, however, a greater percentage of higher frequency non-synonymous SNPs are lost in all populations, likely representing selection against deleterious alleles.

**Figure 4:**
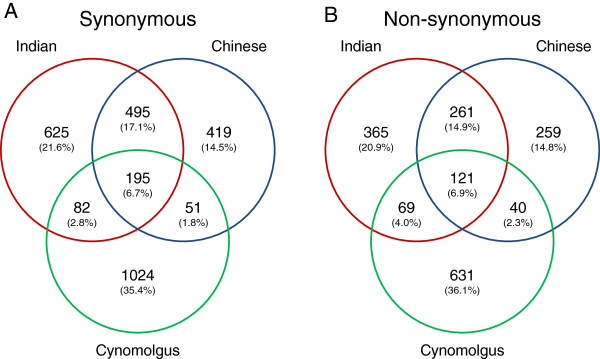
**Venn diagram showing the distribution of SNPs between species. A.** Synonymous changes. **B.** Non-synonymous changes. Only SNPs observed multiple times are included.

These findings extend when population specificity of SNPs is considered (Figure 4). Focusing exclusively on SNPs found in multiple individuals (non-singletons) the percentage of SNPs found in both Indian and Chinese rhesus populations is roughly one third with synonymous SNPs only slightly more likely to be found in both populations compared to non-synonymous SNPs (37.0% and 31.8% respectively). But while synonymous SNPs are more likely to be private to Indian-origin animals (37.9% compared to 25.2% Chinese), non-synonymous SNPs are more often private to Chinese-origin rhesus (41.5% compared to 26.6% Indian). If non-synonymous SNPs are considered to be under greater selective constraint, then these findings are suggestive of either greater constraint in Indian-origin animals (seemingly unlikely) or a recent population expansion in these Indian animals when compared to the Chinese animals. This latter finding is consistent with the allele frequency spectrum data though shares the same caveats with regard to human selective breeding.

Previous studies have demonstrated that cynomolgus macaques share polymorphism with rhesus macaques [28,41]. Using control regions under selective neutrality or presumed constant selective pressures across the species, shared and private polymorphism was used to establish a divergence time of roughly 1.3 MYA and a consistent, if asymmetric, gene flow [42]. Studies focusing on the cytochrome P450 genes, important modulators of xenobiotic metabolism, have shown a relative increase in private polymorphism thought to perhaps represent the effects of differential selective regimes [43]. Interestingly, in GPCRs a greater percentage of non-synonymous SNPs (20.2%) are shared between the species than synonymous SNPs (11.3%). This distinction is further muddied, however, when the two rhesus subpopulations are taken into account. Among synonymous SNPs the majority of shared polymorphisms (59.5%) are shared among cynomolgus macaques and both rhesus subpopulations, compared to only 23.5% of non-synonymous SNPs. The preponderance of shared synonymous SNPs is consistent with previous, smaller-scale, findings on non-coding SNPs [28] and is roughly consistent with expectations under neutrality. The preponderance and distribution of non-synonymous SNPs, however, are perhaps indicative of balanced selection.

Much of these findings have concentrated on general descriptions of the polymorphism profile of the macaque populations. While these results have focused on protein-coding regions more likely under negative selective pressures than previous studies of presumably, or more likely, neutral variation, the results have by and large been the same. To this point, the most notable finding is that non-synonymous polymorphisms seem more likely to be shared between populations than synonymous variation. While informative, general demographic understandings are better approached through neutral variation and that was not the primary purpose here. Rather, the focus of this study was in identifying and understanding likely functionally relevant variation aimed at improving the usage of macaques as biomedical research models. The focus on GPCRs, the most common of druggable targets, belies this goal.

### Distribution of variation

To understand the variation most likely to be functionally relevant in the GPCRs an initial focus was on polymorphism location with regards to secondary structure. Macaque sequences derived from existing annotation coupled with refinements from the consensus resequencing results were aligned with human sequences. Secondary structures for human proteins were pulled from the UniProt database. The consensus macaque sequences were aligned and fixed divergent sites between macaque and humans were mapped onto secondary sequences. In accordance with expectations, fixed synonymous mutations were distributed homogenously across the protein without regard for secondary structure. Non-synonymous differences, however, were non-randomly distributed across the secondary structure. Transmembrane domains were significantly more conserved than either intracellular or extracellular domains. N-terminal and C-terminal domains were the most divergent between taxa and the first and second intracellular domains were the most conserved of the non-transmembrane domains. These findings are consistent with understandings of GPCR structure and function given that transmembrane domains are expected to be under strong functional constraint to maintain secondary structure and hydrophobicity. Extracellular domains mediate ligand binding with functional residues largely spread across the three loops. Intracellular signaling domains are largely mediated through either the C-terminal domain or the third intracellular loop depending on the nature of the particular GPCR and, therefore, divergence in these domains suggests an evolutionary lability to these functions and drives a need for improved understanding.

**Figure 5:**
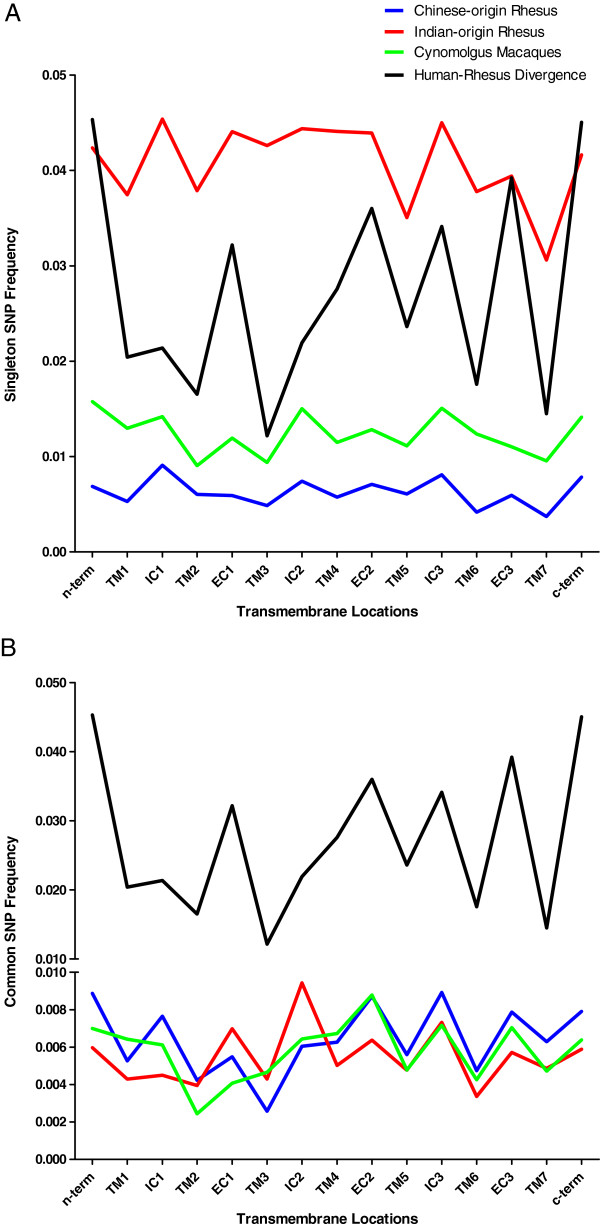
**Frequency of divergence and polymorphism in secondary structure including all seven transmembrane regions, intra and extracellular domain regions. A.** Singleton polymorphisms. **B.** Polymorphisms observed in multiple individuals (common).

As with fixed differences, synonymous SNPs in each of the populations are distributed evenly and consistently across the protein. This distribution, driven by neutral mutation rate and largely unaffected by selection, is also seen in the distribution of singletons across the secondary structure (Figure 5A). In comparison, SNPs that are found in multiple individuals show distribution patterns across the proteins more similar to those seen in divergence with human (Figure 5B). This pattern also holds for human polymorphisms when the cutoff for common SNPs is arbitrarily placed at 1%. Again it is supposed that rare SNPs include many slightly deleterious mutations that are destined to be selected out of the population, while more common polymorphisms show patterns consistent with the effects of selective forces.

This can further be explored through the use of functional prediction algorithms. Three unique algorithms were used to classify each of the macaque non-synonymous changes: PolyPhen-2 [44], SIFT [45], and EvoD [46]. A consensus of these was used to classify non-synonymous SNPs as “ambiguous”, “deleterious”, “likely deleterious”, “likely neutral”, or “neutral” after established methods [47]. Regardless of the frequency of the SNPs, singletons or multiples, the percent identified as damaging was statistically the same (roughly 55%). There was also no difference in the proportion of damaging SNPs within the various populations and subpopulations. This also did not significantly vary based on the secondary structure domain within the protein or on their distribution between subpopulations (Additional file 2: Figure S1 and Additional file 3: Figure S2).

These findings run contrary to what is seen in humans. In humans, as one would predict if these predicted deleterious SNPs are truly damaging, the more common the SNP the less likely it is to be classified as deleterious [47]. Here not only is there not a correlation between frequency and likelihood of being damaging, but there also seems to be no correlation with secondary structure domain. This is despite the fact that there does seem to be a correlation between non-synonymous SNP frequency and domain as predicted by our conceptual understandings of GPCR structure and function. There are several possible explanations for this observed phenomenon. The first and more intriguing is that SNPs being classified as deleterious are perhaps more likely to change protein function but not necessarily in a selectively negative way. Some portion of these SNPs could thus be beneficial and driven to higher frequencies. More likely, however, are much more mundane explanations that these algorithms simply are not designed to work well across species and do not or that the frequencies of alleles observed in these populations are the result of human selective breeding forces in biomedical research colonies and not representative of natural selective effects.

### Functional variation

Regardless, the primary motivation for this study was to understand how functional variation in macaque GPCRs might be used to better understand evolutionary adaptation and the role of macaques as biomedical research models. One question in particular is how variation in human GPCRs might compare to variation in their macaque orthologs and whether functional effects in humans could be better understood or possibly even modeled in macaques. To investigate this, human polymorphisms with frequencies greater than one-half of one percent (0.5%) were drawn from dbSNP. While arbitrary, these criteria ensured the validity of the SNP and at least a modicum of data. It is important to note, however, that human SNPs were not chosen by frequencies in specific subpopulations and there are notable issues of ascertainment bias still present in the human data set. Human SNPs were then mapped to secondary structures following the same methodologies of the macaque polymorphisms and the two data sets were compared.

Somewhat unexpectedly, though perhaps not in retrospect, nine recurrent mutations (Table 3) were identified. These mutations are present in both humans and macaques. Only SNPs present in multiple macaque animals were included and the animals sharing these “human” alleles were different so it is reasonably certain that they represent real macaque SNPs. These polymorphisms do not represent true trans-species polymorphisms of a shared origin, but rather are recurrent mutations at the same position. It remains unclear if this is due simply to chance or if there are similar underlying evolutionary pressures. While there is neither functional information nor phenotypic associations with these SNPs in humans, it is perhaps interesting to note that consensus predications from PolyPhen-2, SIFT, and EvoD show six of nine as “deleterious” or “likely deleterious”. In comparison there are only five instances where the same ancestral amino acid was mutated to two different amino acids in human and macaques (Table 4). In these cases, the majority of changes are categorized as neutral, though in MRGPRX1 both human, Arg55Leu, and macaque, Arg55Cys, polymorphisms are predicted to be deleterious.

**Table 3:** Recurrent mutations

					**Human**			**Macaque**			
**Gene**	**AA position**	**AA1**	**AA2**	**Consensus prediction**	**GRCh37.p5**	**dbSNP**	**Human MAF**	**rheMac2**	**Chinese MAF**	**Indian MAF**	**Fascicularis MAF**
FZD6	664	A	E	Deleterious	chr8:104343607	rs12549394	0.02	chr8:105850125	0.00	0.00	0.10
GPR19	116	V	I	Likely deleterious	chr12:12815037	rs41276680	0.01	chr11:13015710	0.02	0.00	0.22
GPR44	204	V	A	Likely neutral	chr11:60620585	rs2467642	0.01	chr14:13261124	0.07	0.02	0.83
GPR78	342	R	H	Deleterious	chr4:8589023	rs9685931	0.11	chr5:197204	0.05	0.00	0.00
GPR98	194	P	H	Deleterious	chr5:89920969	rs61745498	0.02	chr6:86859930	0.00	0.05	0.00
GPR146	266	V	M	Deleterious	chr7:1097947	rs55677825	0.01	chr3:38980764	0.14	0.00	0.00
GPR153	209	R	H	Neutral	chr1:6313938	rs12735670	0.31	chr1:9260385	0.10	0.00	0.00
GPR156	798	R	H	Likely neutral	chr3:119885931	rs115365859	0.01	chr2:40203636	0.00	0.00	0.14
MRGPRX3	198	L	R	Likely deleterious	chr11:18159342	rs28482781	0.02	chr14:53770726	0.07	0.00	0.00

AA: Amino acid, MAF: Minor allele frequency.

**Table 4:** Shared amino acid mutations

		**Human**								**Macaque**				
**Gene**	**AA Position**	**AA1**	**AA2**	**Consensus Prediction**	**GRCh37.p5**	**dbSNP**	**Human MAF**	**AA1**	**AA2**	**Consensus Prediction**	**rheMac2**	**Chinese MAF**	**Indian MAF**	**Fascicularis MAF**
DRD5	330	P	Q	Likely Neutral	chr4:9784642	rs1800762	0.03	P	L	Neutral	chr5:4664928	0.12	0.00	0.00
GPR78	318	R	C	Likely Neutral	chr4:8588950	rs61746144	0.01	R	H	Neutral	chr5:197276	0.00	0.00	0.17
HTR1E	208	A	T	Likely Deleterious	chr6:87725674	rs3828741	0.01	A	S	Likely Neutral	chr4:82975291	0.00	0.03	0.00
MRGPRX1	55	R	L	Deleterious	chr11:18956168	rs55954376	0.01	R	C	Deleterious	chr14:52926663	0.00	0.24	0.00
P2RY4	168	V	M	Likely Neutral	chrX:69478973	rs1152186	0.05	V	A	Likely Neutral	chrX:69226991	0.07	0.00	0.00

AA: Amino acid; MAF: Minor allele frequency.

In total, 128 instances were identified in which “common” human variation was found in the same gene and protein secondary structure domain as “common” macaque variation (Additional file 4: Table S2). These spanned 99 distinct genes or roughly one-third of the GPCRs resequenced in this study. Although the majority of these were located in either the N-terminal (38%) or C-terminal (29%) domains, shared variation was found in every secondary structure domain. The third intercellular domain, often associated with the signaling functions of the GPCRs, had the third greatest amount of shared variation (11%). Further, more than half of all SNPs identified this way in macaques are predicted to be “deleterious” or “likely deleterious”.

Of these, it is useful to highlight some specific examples. The known parallel functional variation between human and rhesus macaques in *OPRM1* is recapitulated here. In the N-terminal domain of the mu-opioid receptor, two human polymorphisms C17T (Ala6Val) and A118G (Asn40Asp) show parallel in vitro functional effects with the Indian rhesus macaque C77G (Pro26Arg) mutation [48,49] as well as parallel phenotypic associations with alcohol consumption and response to naltrexone [50-52]. This parallel function has already proven to be a useful tool in elucidating the role of the mu-opioid receptor in alcoholism. Prior to the rhesus macaque studies, human work had been inconclusive despite a relatively large number of studies [53,54]. This variability across studies, inherent in human research due to genetic and environmental heterogeneity, could be quickly and simply teased apart using carefully selected and managed non-human primate models.

In another example, early studies have tentatively linked human variation in *ADRA1A* with complex pain and fibromyalgia [55,56] and specific variation in the third intracellular domain, Gly247Arg, with receptor pharmacology [57]. While not identical, one common polymorphism is found in the third intracellular loop in macaques, Arg266Leu, with predicted deleterious effects. Two polymorphisms are also found in the C-terminal domain, Lys349Arg and Arg405His, where associations have also been seen in humans.

Several other human variants with putative associations also have possible homologs in macaques. In the oxytocin receptor (OXTR), Ala218Thr has been associated with emotional empathy in humans [58], while Ser224Cys, in the same receptor domain, is a common polymorphism in Chinese-origin rhesus and cynomolgus macaques. Somatostatin receptor 4 (SSTR4) variation, Phe327Ser, has been associated with response to colorectal cancer treatment in humans [59], and rhesus macaques and cynomolgus macaques harbor common polymorphisms Ala357Asp and Met360Val, respectively. Variation in follicle-stimulating hormone receptor (*FSHR*) and histamine receptor H4 (*HRH4*) have been associated with polycystic ovarian syndrome [60] and breast cancer [61] respectively and likewise similar polymorphisms may be observed in macaques.

These examples only scratch the surface with the focus here on common human variation, not pathogenic variation. It is possible that there are additional examples of pathogenic variation that is modeled in macaques or human variation that simply has yet to be recognized as pathogenic due to the vagaries of human research. Common macaque polymorphism may illuminate the functional relevance of human variation even in the absence of known human associations. Variation found in the same genes and secondary structures in humans and macaques offer potentially informative targets for studies of functionally similar, though evolutionarily distinct, variation across species and for the improvement of understanding the molecular underpinnings of disease.

## Conclusions

Drug discovery and translational medicine benefit from strong animal models. For too long poor animal models have led researchers down the wrong paths, leading, perhaps, to novel understandings and interesting results, but not to improved treatments in humans that have been promised. In part, the scientific community has been playing the cards it was dealt, too quick to believe that shared phenotypes implied a shared molecular basis. Now, however, the revolution in sequencing technologies allows us to look closer at the molecular basis of disease than has ever been possible and, in doing so, we can more easily identify when shared phenotypes do share molecular bases and when they do not. Moreover, we can identify where similar molecular and genetic foundations exist, but do not lead to the same phenotypic effects.

Non-human primates have long been known to share genetic and physiological similarities with humans. This has made them the gold standard for preclinical research, though one for which it has not always been clear if the benefits outweighed the price. By better understanding the genetics of non-human primates we lay clear the benefits, demonstrating where genetic similarities exist with humans and where non-human primates are most likely to be beneficial. We also develop tools for maximizing the utility of non-human primates, ensuring that when they are used as biomedical research models they are used appropriately and result in the greatest power.

Here we catalog the polymorphism in the GPCRs of rhesus macaques of Indian and Chinese origin and Mauritian cynomolgus macaques. Together these species represent the most commonly used non-human primate biomedical research models and the genes represent the single largest family of drug targets. This information can be used going forward to develop improved animal models and to better understand gene-phenotype associations. By improving our animal models we improve the ability of our science to be translational and ultimately to bring basic research to bear on issues of human health.

## Methods

### Ethics statement

Blood draws for the isolation of genomic DNA for animals used in this study were done during routine preventative health care by trained veterinary phlebotomists within the NEPRC Division of Veterinary Resources. All animals were maintained in accordance with the guidelines of the Harvard Medical School Standing Committee on Animals and the Guide for Care and Use of Laboratory Animals of the Institute of Laboratory Animal Resources, National Research Council.

### Animals and genomic DNA

Blood from 32 cynomolgus macaques (*Macaca fascicularis*), 44 Indian-origin rhesus macaques (*M. mulatta*) and 20 Chinese-origin rhesus macaques was collected in EDTA vacutainer tubes (BD, Franklin Lakes, NJ) during standard preventative health care. Genomic DNA was isolated using DNeasy Blood and Tissue Kit protocols (Qiagen, Valencia, CA). 17 Indian-origin rhesus were born at the New England Primate Research Center (NEPRC), 13 born at the Oregon National Primate Research Center (ONPRC) and 14 born at the Caribbean Primate Research Center (CPRC). Chinese-origin rhesus were purchased from Charles River Laboratories. All animals had been housed at the NEPRC for at least three years prior to blood draws obtained for this study. Cynomolgus macaques, also housed at the NEPRC a minimum of three years at the time of study, were purchased from Charles River Laboratories and were of purported Mauritian origin.

### Target capture and next generation sequencing

A custom SureSelectXT (Agilent Technologies, Santa Clara, CA) library was designed using GPCRs from both the human and rhesus macaque genomes as baits. While ideally the rhesus genome should be sufficient and best for capture of macaque targets, annotation remains incomplete and gaps persist. These problems are not present to the same degree in the human genome and the flexibility of the technology can support the divergence between humans and old world monkeys [62].

Following capture, sequencing libraries were prepared using the SureSelectXT library preparation kits and protocols with barcodes for 24x multiplexing (Agilent Technologies, Santa Clara, CA). Prior to sequencing, libraries undergo quality control using an Agilent Bioanalyzer 2100 (Agilent Technologies, Santa Clara, CA). Next generation sequencing was performed on HiSeq 2000 (Illumina Inc, San Diego, CA) using a 50 bp single end read protocol. Target enrichment, library preparation, and next generation sequencing was performed at the Biopolymers Facility, Department of Genetics, Harvard Medical School, Boston, MA.

### Data analysis

Initial data analysis was processed through DNAnexus (DNAnexus Inc., Mountain View, CA). All reads were aligned to the rhesus genome (MGSC Merged 1.0/rheMac2). Using Geneious version 6.0.5, (created by Biomatters, San Francisco, CA) additional alignments using 'bowtie’ and 'velvet’ were implemented though they did not show meaningful differences. Average read depth in coding regions among animals was >100x, ranging from >200x to 50x. Variability between samples is likely due to effects of multiplexing as well as sample quality. Read depth was also notably greater in coding sequences compared to untranslated regions, presumably due to poorer capture efficiency in the UTRs as a result of greater sequence divergence.

The “nucleotide-level variation” analysis pipeline implemented in DNAnexus was used to identify and call polymorphic sites in each individual animal. Allelic variation was called using a Bayesian model which incorporates quality scores, read/reference mismatches, and SNP rate priors [63]. It is anticipated that at these read depths SNP identification coverage approaches full sensitivity [64].

Human orthologs were identified using Homologene and Ensembl and were aligned to the hand curated rhesus genes. Divergence values were calculated using Perl scripts developed in-house. Secondary structure, notably including the positions of transmembrane domains, were determined for the human orthologs using information gathered from the UniProt database [65] and transliterated to the aligned rhesus ortholog.

Non-synonymous macaque polymorphisms were mapped onto orthologous human sequences and run through predictive algorithms for evaluating their impact on protein function. PolyPhen-2 [44] and SIFT [45] were evaluated as well as their evolutionarily-balanced implementation [47] and the EvoD algorithm [46]. Transliteration posed difficulties first due to poor or incomplete annotation in the rhesus macaque genome and second due to actual biologically meaningful divergence between the species. Also, because many of these algorithms make use of multi-species conservation in their implementation, it is unclear how this may affect regions “known” to be divergent between the taxa. Because of these issues a conservative approach was taken whereby the predictive algorithms were run only on variation where the mutated amino acid was unambiguously present and conserved in humans.

## Competing interests

The authors declare that they have no competing interests.

## Authors’ contributions

EJV and GMM conceived the study. EJV designed the study. DBG, LMO, JMW, and EJV performed data analysis and bioinformatics analyses. DBG, LMO, and EJV drafted the manuscript. All authors have read and approve of the final manuscript.

## Supplementary Material

Additional file 1: Table S1 All SNPs identified by this survey. Click here for file

Additional file 2: Figure S1 Consensus functional prediction of SNPs in macaques by secondary structure domain. A. Singleton polymorphisms. B. Polymorphisms observed in multiple individuals (common). Click here for file

Additional file 3: Figure S2 Venn diagram with pie charts showing distribution of consensus functional predictions of SNPs in macaques. Click here for file

Additional file 4: Table S2 Common variation in human and macaque within a secondary structure domain. Click here for file
