# Supplementary figures and images for: Large-scale polymorphism discovery in macaque G-protein coupled receptors
Source: BMC Genomics. 2013 Oct 11;14:703. doi: 10.1186/1471-2164-14-703 (PMC3907043; doi:10.1186/1471-2164-14-703)

## Singleton

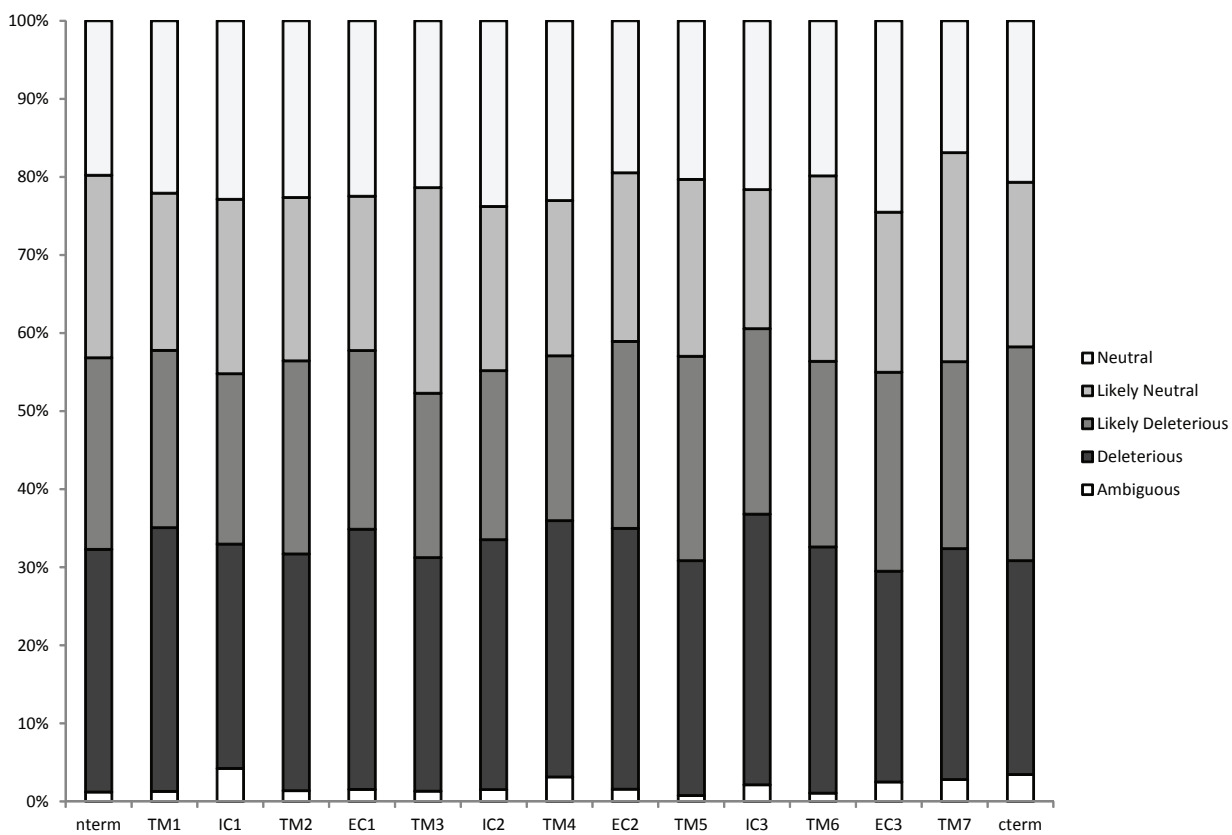

## Common

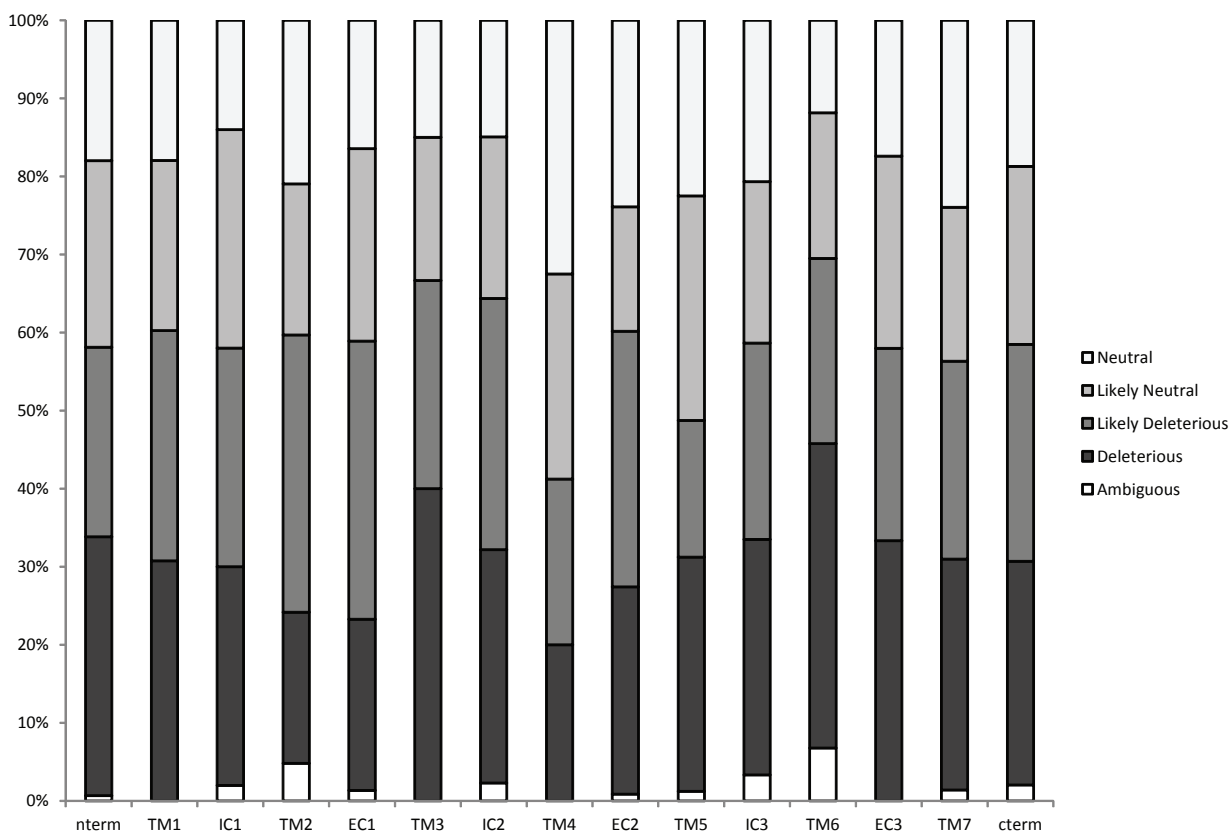

Supplement: Additional file 2: Figure S1 — Consensus functional prediction of SNPs in macaques by secondary structure domain. A. Singleton polymorphisms. B. Polymorphisms observed in multiple individuals (common). [file 1471-2164-14-703-S2.pdf]

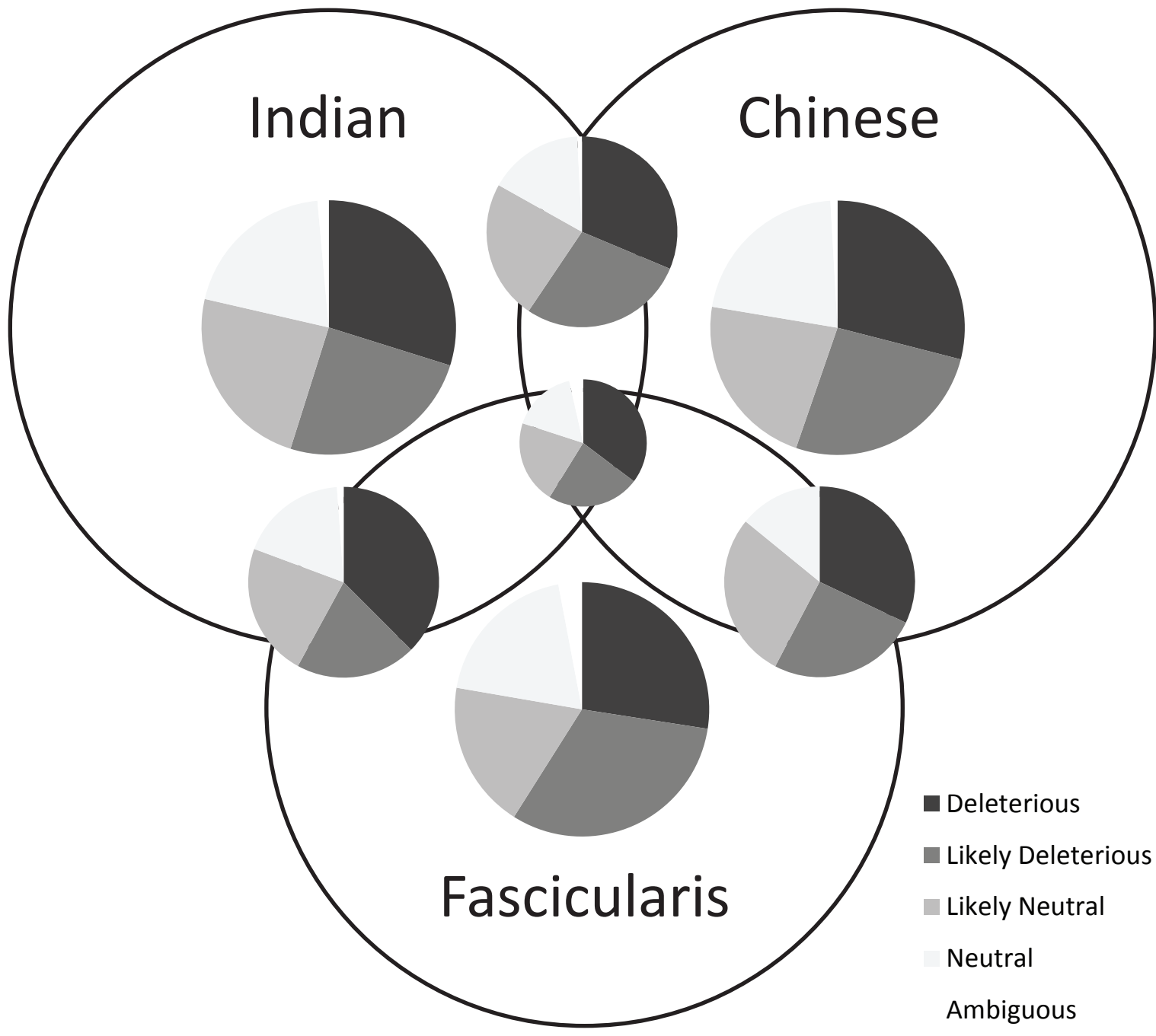

Supplement: Additional file 3: Figure S2 — Venn diagram with pie charts showing distribution of consensus functional predictions of SNPs in macaques. [file 1471-2164-14-703-S3.pdf]
